# Sustained Release of Co-Amorphous Matrine-Type Alkaloids and Resveratrol with Anti-COVID-19 Potential

**DOI:** 10.3390/pharmaceutics14030603

**Published:** 2022-03-10

**Authors:** Dandan Hu, Xin Chen, Duanxiu Li, Hailu Zhang, Yanwen Duan, Yong Huang

**Affiliations:** 1 Xiangya International Academy of Translational Medicine, Central South University, Changsha 410013, China; dandanhu@yeah.net (D.H.); xchen2016@sinano.ac.cn (X.C.); ywduan66@csu.edu.cn (Y.D.); 2 Laboratory of Magnetic Resonance Spectroscopy and Imaging, Suzhou Institute of Nano-Tech and Nano-Bionics, Chinese Academy of Sciences, Suzhou 215123, China; dxli2016@sinano.ac.cn (D.L.); hlzhang2008@sinano.ac.cn (H.Z.); 3 Guangdong Institute of Semiconductor Micro-Nano Manufacturing Technology, Foshan 528200, China; 4 Hunan Engineering Research Center of Combinatorial Biosynthesis and Natural Product Drug Discovery, Changsha 410011, China; 5 National Engineering Research Center of Combinatorial Biosynthesis for Drug Discovery, Changsha 410011, China

**Keywords:** co-amorphous systems, sustained-release, COVID-19, matrine-type alkaloids, resveratrol

## Abstract

Matrine (MAR), oxymatrine (OMAR), and sophoridine (SPD) are natural alkaloids with varying biological activities; matrine was recently used for the treatment of coronavirus disease 2019 (COVID-19). However, the short half-lives and rapid elimination of these matrine-type alkaloids would lead to low oral bioavailability and serious side effects. Herein, resveratrol (RES) was selected as a co-former to prepare their co-amorphous systems to improve the therapeutic index. The formation of co-amorphous MAR-RES, OMAR-RES, and SPD-RES was established through powder X-ray diffraction and modulated temperature differential scanning calorimetry. Furthermore, Fourier transform infrared spectroscopy and NMR studies revealed the strong molecular interactions between resveratrol and these alkaloids, especially OMAR-RES. Matrine, oxymatrine, and sophoridine in the co-amorphous systems showed sustained release behaviors in the dissolution experiments, due to the recrystallization of resveratrol on the surface of co-amorphous drugs. The three co-amorphous systems exhibited excellent physicochemical stability under high relative humidity conditions. Our study not only showed that minor structural changes of active pharmaceutical ingredients may have distinct molecular interactions with the co-former, but also discovered a new type of sustained release mechanism for co-amorphous drugs. This promising co-amorphous drug approach may present a unique opportunity for repurposing these very promising drugs against COVID-19.

## 1. Introduction

The coronavirus disease 2019 (COVID-19) pandemic caused by severe acute respiratory syndrome coronavirus 2 (SARS-CoV-2) has led to over 5 million deaths worldwide as of early December 2021 [1]. The rapidly developed various types of vaccines against SARS-CoV-2, such as the inactivated virus COVID-19 vaccine CoronaVac and the Pfizer-BioNTech and Moderna COVID-19 mRNA vaccines, have shown effectiveness against SARS-CoV-2 spread and significantly reduced the severity and death rate among more vulnerable populations [2]. However, the rapid evolving variants of SARS-CoV-2 may render these vaccines less effective. Drug reposition is a rapid approach to develop life-saving medications against COVID-19 in a global health crisis. For example, high throughput screenings of various drug collections have yielded multiple drug leads against viral replication of SARS-CoV-2, including PIKfyve kinase inhibitor apilimod, multiple cysteine protease inhibitors, as well as farnesyltransferase inhibitor tipifarnib and phosphatidylinositol 3-kinase inhibitor omipalisib [3,4].

**Scheme 1:**
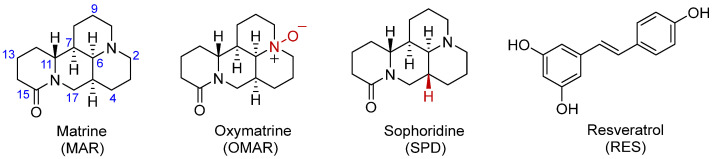
Molecular structures of MAR, OMAR, SPD, and RES.

Natural products have great potential to treat SARS-CoV-2 [5], among which plant-derived medicines, including the lung cleansing and detoxifying decoction with 21 ingredients, have been used against COVID-19 in China [6,7]. Matrine (MAR), a representative quinolizidine alkaloid from *Sophora flavescens Ait*, has been used to treat chronic hepatitis and enteritis for years in China (Scheme 1, Table S1) [8]. Impressively, matrine was used to treat 40 COVID-19 patients, in addition to other recommended treatment regimens, such as antiviral drugs abidol or lopinavir and ritonavir in early 2020 [9]. These treatments markedly reduced the fibrotic and grid-like lesions in the lungs of patients by chest computed tomography scan and cleared SARS-CoV-2 from patients within 10 days of the combination therapy. Furthermore, the mortality rate among hospitalized COVID-19 patients strongly correlated with the autoimmunity level to annexin A2 [10], suggesting its importance in SARS-CoV-2 pathogenicity [11]. Matrine was shown to directly target annexin A2 by a photo-affinity labeling approach [12], while very recent network pharmacological studies suggested that matrine may also have varying targets in cellular signaling pathways [13,14,15]. Oxymatrine (OMAR) and sophoridine (SPD, Scheme 1) are matrine-type alkaloids isolated from *S. flavescens Ait* with promising biological activities [16,17], including antiviral activity [18,19]. Resveratrol (RES) is a plant polyphenol, which has also been used to treat COVID-19 patients with mild symptoms or as adjuvant supplements due to its anti-SARS-CoV-2 and anti-inflammatory activities [20,21].

However, matrine was given to COVID-19 patients via intravenous infusion [9], suggesting its poor pharmacokinetic properties, which may result in poor patient compliance and persistence, as well as unsatisfactory outcomes. Several studies of oral administration of matrine, oxymatrine, and sophoridine in model animals showed that their absolute bioavailability was 17.1% [22], 6.79% [23], and 2.32% [24], with half-lives of 1.53 ± 0.53 [22], 6.45 ± 1.07 [23], and 4.67 ± 1.50 h [24], respectively. Drug co-amorphization is an emerging technology to improve the physicochemical properties of oral drugs [25], which is a single-phase amorphous system formed between an active pharmaceutical ingredient and one or more low molecular weight compounds named co-formers [26]. For example, it may improve the bioavailability of poorly water-soluble drugs by increasing their solubilities, such as indomethacin and apigenin [27,28]. However, co-amorphization of drugs would not always enhance their solubilities or dissolution rates. The solubility of certain drugs in co-amorphous systems may be impacted by many factors, including co-amorphization, the solubilities of co-formers, and intermolecular interactions between drugs and co-formers. Hence, the solubilities and dissolution rates of drugs in co-amorphous systems may be decreased, which provides an excellent opportunity for the sustained release of drugs. We and others have recently discovered that certain co-amorphous drug delivery systems exhibited sustained release behaviors, including lurasidone hydrochloride, indomethacin/paracetamol [29,30], sinomenine/phenolic acids, and sinomenine/nonsteroidal anti-inflammatory drugs indomethacin, naproxen, and sulindac [31,32]. Therefore, we envision that a new sustained release-based strategy for matrine-type alkaloids is needed to realize their full translational potential in COVID-19 therapy. 

In this study, we test the hypothesis if resveratrol can be used as a new co-former for the formation of co-amorphous drug systems with matrine, oxymatrine, and sophoridine, due to the potential synergistic effects against SARS-CoV-2. These new solid formulations have been prepared and systematically characterized by powder X-ray diffraction (PXRD), Fourier transform infrared spectroscopy (FTIR), modulated temperature differential scanning calorimetry (mDSC), scanning electron microscopy (SEM), and nuclear magnetic resonance (NMR) spectroscopy. Further, they showed distinct sustained release behaviors, which are likely correlated to varying molecular interactions of matrine, oxymatrine, and sophoridine with resveratrol, as well as the unexpected recrystallization of resveratrol from the co-amorphous systems on the surface of drug tablets. Our study not only results in three new co-amorphous drug systems with great anti-COVID-19 potential, but also suggests a promising translational route for natural products from traditional Chinese medicines.

## 2. Materials and Methods

### 2.1. Materials

All compounds used in this study were from commercial sources and used as received, including matrine (≥98%), oxymatrine (≥96%), and sophoridine (≥99%) (Bide Pharmatech, Shanghai, China) and resveratrol (≥99%) (Aladdin, Shanghai, China). All other chemical reagents were from Sinopharm Chemical Reagent (Shanghai, China). 

### 2.2. Preparation of the Co-Amorphous Systems

The solvent evaporation method was applied for preparation of the co-amorphous systems. Briefly, the mixture of crystalline matrine and crystalline resveratrol with molar ratio of 1:1 (0.2 mmol) was dissolved in 5 mL of methanol, followed by rotary vacuum evaporation at 50 °C. The resulting samples were collected and dried under vacuum at 30 °C for 24 h to remove the residual solvent and stored under a dry environment at room temperature for further investigation. The co-amorphous systems of oxymatrine and sophoridine with resveratrol were similarly prepared. 

### 2.3. Solid-State Characterization

#### 2.3.1. PXRD 

The PXRD spectra of all samples were recorded on a Bruker D8 Advance X-ray powder diffractometer with a Cu Kα radiation (λ = 1.5406 Å) source at 25 °C. The process parameters were set at a tube voltage of 40 kV and amperage of 40 mA. The tested samples were placed in monocrystalline sample disks. For each sample, the data were collected in a 2θ range of 3–40° with a step length of 0.0194° at a scanning speed of 0.1 s/step.

Mercury 3.8 was used to calculate the simulated PXRD pattern of oxymatrine trihydrate based on its single crystal data (No. 2058431) downloaded from the Cambridge Crystallographic Data Centre.

#### 2.3.2. mDSC

The thermodynamic behaviors of all crystalline samples and three co-amorphous samples were characterized using a TA Discovery 250 differential scanning calorimeter (TA Instruments, New Castle, DE, USA). Calibration of the instrument was carried out using indium as a standard, and sapphire disks were used for T_zero_ calibration. Each sample (3–5 mg) was placed in an aluminum pan, followed by heating at a scan rate of 2 K·min^−1^ under a dry nitrogen purge (≥99.999%) at a flow rate of 50 mL·min^−1^. The samples were scanned from 30 to 200 °C (oxymatrine was scanned from 30 to 230 °C; resveratrol was scanned from 30 to 300 °C) with an amplitude of 1.00 °C and a period of 60 s.

#### 2.3.3. FTIR 

An IR spectrophotometer (Nicolet Impact 410, Thermo Fisher, Waltham, MA, USA) was employed to record the FTIR spectra of all the samples. About 2 mg of each sample was mixed with 200 mg of potassium bromide (spectral grade purity) and compressed into tablets at room temperature. The FTIR spectra data were collected over the wavenumber range of 400–4000 cm^−1^ with a distinguishing ability of 4 cm^−1^.

### 2.4. NMR 

The ^1^H-NMR and ^13^C-NMR spectra of crystalline matrine, oxymatrine, sophoridine, resveratrol, and the three co-amorphous samples were recorded using an AVANCE III 400 MHz NMR spectrometer with DMSO-*d*_6_ as a solvent at 25 °C.

### 2.5. Dissolution Experiments 

#### 2.5.1. Equilibrium Solubility Determination 

Equilibrium solubility experiments of crystalline forms of matrine, oxymatrine, sophoridine, and the three co-amorphous forms were performed. Excess quantity of each sample was weighed and added into a vial containing 1 mL phosphate buffer saline (PBS) (pH 6.8). The obtained suspension was stirred at 500 rpm in a water bath for 72 h with temperature of 37 ± 0.5 °C and filtered by a 0.22 μm polycarbonate filter. Then, the filtrate was diluted with fresh PBS to an appropriate concentration for high-performance liquid chromatography (HPLC) analysis. Each experiment was repeated three times.

#### 2.5.2. In Vitro Release Experiment 

The release behaviors of matrine, oxymatrine, and sophoridine, as well as the co-amorphous samples, were investigated by the paddle method. The powder samples contained 50 mg of matrine, oxymatrine, and sophoridine were accurately weighed and compressed at a pressure of 6 MPa to obtain tablets with 8 mm diameter for the release experiments. The release experiments were carried out in a dissolution tester (RC-6, Tianguang, Tianjin, China) at a rotational speed of 50 rpm in PBS (100 mL, pH = 6.8) as a dissolution medium at 37 ± 0.5 °C. At the predetermined time points (1, 5, 10, 20, 30, 60, 120, 240, 360, and 720 min), the dissolution medium (1 mL) was acquired and the equal volume of fresh PBS was supplemented. The acquired sample was next filtrated through a polycarbonate filter (0.22 μm), diluted with fresh PBS, and analyzed by HPLC. Triplicate experiments were conducted in parallel. At the end of release experiments, the tablets after release tests were collected and dried at room temperature. The solid samples on the surface and the core of the tablets were separately used for PXRD characterization.

Waters e2695 HPLC (ACQUITY Arc, Milford, MA, USA) equipped with a 2998 photodiode array detector and an AQ-C18 column (250 mm × 4.6 mm, 5 μm) was used. Matrine, oxymatrine and sophoridine were detected at UV absorption wavelengths of 220 nm. The mobile phase consisted of water (with 0.1% formic acid) and acetonitrile (50:50 *v*/*v*) at flow rate of 1 mL·min^−1^.

### 2.6. Scanning Electron Microscopy 

The surface morphology of the tablets before and after release tests were characterized by scanning electron microscopy (SEM) (FEI Czech Republic s.r.o. Quanta FEG 250, Columbia, MD, USA). The samples were mounted on aluminum stubs with double-sided sticky discs of conductive carbon, and then coated with gold, followed by microscopical scanning.

### 2.7. Physical Stability

All co-amorphous powders were exposed in desiccators contained anhydrous silica gel (low humidity condition) at 25 °C and 40 °C for physical stability evaluation. In addition, their physical stabilities were also studied at 25 °C under a saturated sodium chloride solution atmosphere (75% relative humidity (RH)) [33]. The powder samples were collected at a preset time and measured by PXRD. The physical stability of the three co-amorphous systems against pressure was also investigated under a pressure of 6 MPa for 10 min, resulting in the formation of a tablet. Each tablet was then crushed and characterized by PXRD.

## 3. Results and Discussion

### 3.1. The Design and Preparation of Co-Amorphous Drug Systems of Matrine, Oxymatrine, and Sophoridine with Resveratrol and Their PXRD Characterization

**Figure 1:**
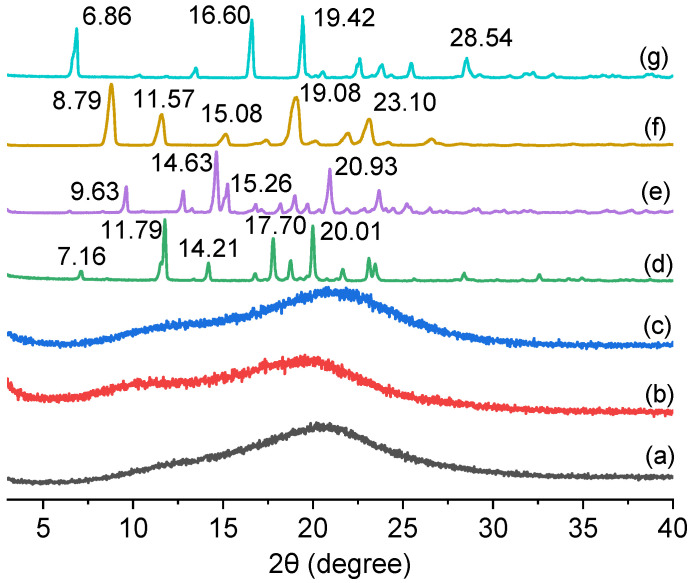
The PXRD patterns of co-amorphous forms of MAR-RES (a), OMAR-RES (b), and SPD-RES (c), as well as crystalline forms of matrine (d), oxymatrine (e), sophoridine (f), and resveratrol (g).

We selected resveratrol as the co-former for the amorphization of matrine, oxymatrine, and sophoridine (Figure 1). In addition, the three quinolizidine alkaloids share the same structure scaffold, since oxymatrine is the *N*-oxide of matrine, while sophoridine is the C-5 epimer of matrine. Although co-amorphous systems have become a promising approach in drug formulation [26,27], to our knowledge, many previously studied systems include a single small molecule drug with one or multiple co-formers. The study of the molecular interactions in these systems have largely focused on the varying interactions and physicochemical properties of drugs and their co-formers, including amino acids, dipeptides, and phenolic acids, among others [34,35,36]. The high structure similarity of matrine, oxymatrine, and sophoridine provides a rare opportunity to study their molecular interactions with the single co-former resveratrol, thus potentially offering new insights of the co-amorphous drug systems.

We adopted the solvent evaporation method to prepare the co-amorphous systems of matrine, oxymatrine, and sophoridine with resveratrol. We next evaluated the PXRD patterns of the prepared solids, including MAR-RES, OMAR-RES, and SPD-RES, through the powder X-ray diffraction analysis, using their respective crystalline forms as controls. For the crystalline forms of matrine, oxymatrine, sophoridine, and resveratrol (Figure 1d–g), characteristic diffraction peaks ((7.16°, 11.79°, 14.21°, 17.70°, and 20.01°), (9.63°, 14.63°, 15.26°, and 20.93°), (8.79°, 11.57°, 15.08°, 19.08°, and 23.10°) and (6.86°, 16.60°, 19.42°, and 28.54°), respectively) appeared at 2θ over 3–40°. The crystalline oxymatrine was confirmed as a trihydrate, since its PXRD pattern was in line with the simulated one using the single crystal data of oxymatrine trihydrate (Figure S1) [37]. In contrast, the three solid samples of matrine, oxymatrine, and sophoridine that co-evaporated with resveratrol exhibited a typical halo with no characteristic peaks (Figure 1a–c), suggesting that all of them were transformed into an amorphous state. However, we were unable to prepare the amorphous forms of matrine, oxymatrine, sophoridine, and resveratrol under the same condition (Figure S2). The PXRD pattern of evaporated oxymatrine exhibited certain differences with its original crystalline form, indicating a polymorphism (oxymatrine monohydrate) formed after treatment (Figure S2c–d) [37], while other parent materials evaporated alone showed no phase transition.

### 3.2. mDSC Study of the Amorphous MAR-RES, OMRA-RES, and SPD-RES Samples

**Figure 2:**
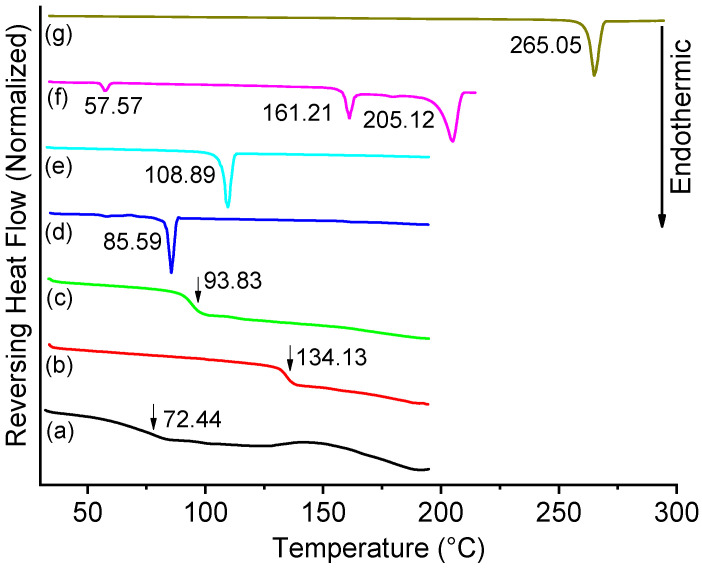
The mDSC curves of co-amorphous forms of MAR-RES (a), OMAR-RES (b), and SPD-RES (c), as well as crystalline forms of matrine (d), oxymatrine (e), sophoridine (f), and resveratrol (g).

In order to investigate the formation of co-amorphous systems, the mDSC curves of the amorphous MAR-RES, OMRA-RES, and SPD-RES samples were obtained by mDSC, along with their individual crystalline form as controls (Figure 2). The crystalline forms of matrine, sophoridine, and resveratrol exhibited endothermic melting peaks at 85.59 °C, 108.89 °C, and 265.05 °C, respectively. Crystalline oxymatrine manifested three endothermic peaks at 57.57 °C, 161.21 °C, and 205.12 °C, respectively [38]. The first endothermic peak corresponded to the phase transition from trihydrate to monohydrate of oxymatrine and the second endothermic peak suggested that the oxymatrine monohydrate transformed into anhydrous oxymatrine. The third endothermic peak was the melting peak of anhydrous oxymatrine [37]. Different from their crystalline counterparts, the co-evaporated samples of MRA-RES, OMAR-RES, and SPD-RES displayed single glass transition temperature (*T*_g_) at 72.44 °C, 134.13 °C, and 93.83 °C, respectively, which supports the formation of homogeneous co-amorphous systems. Obviously, co-amorphous OMAR-RES showed much higher *T*_g_, compared to the other two co-amorphous systems, indicating that it may hold stronger intermolecular interactions.

### 3.3. FTIR

**Figure 3:**
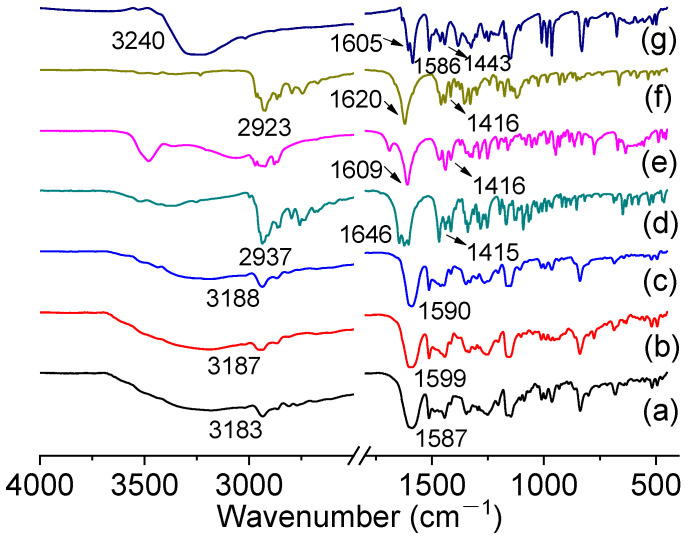
The FTIR spectra of co-amorphous forms of MAR-RES (a), OMAR-RES (b), and SPD-RES (c), as well as crystalline forms of matrine (d), oxymatrine (e), sophoridine (f), and resveratrol (g).

FTIR is an analytical technique used to study the interactions among drugs and co-formers in co-amorphous drug systems, due to its ability to detect characteristic vibrations of chemical bonds by inelastic collisions between tested samples and infrared light. Therefore, the FTIR spectra of co-amorphous MAR-RES, OMRA-RES, and SPD-RES, as well as their crystalline forms and physical mixtures were obtained (Figure 3 and Figures S3–S10). The crystalline matrine showed a typical C=O stretching vibration (*ν*(C=O)) of the lactam functional group at 1646 cm^−1^ and a C–H stretching vibration from methylene groups in 2937 cm^−1^ (Figure 3d). Its characteristic C–N stretching vibration (*ν*(C–N)) peaks appeared at 1415 cm^−1^, consistent with the previous report [39]. The crystalline sophoridine exhibited the similar FTIR spectrum with matrine, with representative peaks of *ν*(C=O) and *ν*(C–N) in 1620 cm^−1^ and 1416 cm^−1^, respectively (Figure 3f). In contrast, the crystalline oxymatrine had peaks in 2286 cm^−1^ and 1609 cm^−1^ [40], which are attributed to N^+^–O^−^ stretching vibration (*ν*(N^+^–O^−^)) and *ν*(C=O) of the lactam, respectively (Figure 3e and Figure S4). The crystalline resveratrol manifested broad stretching vibration signals of the phenolic hydroxyl groups (*ν*(O–H)) at 3240 cm^−1^, and representative peaks at 1605 cm^−1^, 1586 cm^−1^, and 1443 cm^−1^ belonging to C–C stretching vibrations and planar C–C–H deformation vibrations of the two benzene moieties (Figure 3g). A superposition of the spectra of the pure components was observed in the three physical mixtures, indicating that no intermolecular interactions occurred between the individual components (Figure S10).

However, in the FTIR spectra of all co-amorphous samples, their characteristic peaks became broad and blunt compared to those of their crystalline counterparts (Figure 3a–c). For example, the sharp peak of *ν*(O–H) of resveratrol at 3240 cm^−1^ was shifted to 3183 cm^−1^, while the *ν*(C=O) peak of matrine was shifted from in 1646 cm^−1^ to 1587 cm^−1^. The obvious broadened and shifted stretching vibration peaks of both matrine and resveratrol indicate that the intermolecular interactions may exist between matrine and resveratrol. Similar FTIR changes could also be observed in co-amorphous OMAR-RES and SPD-RES (Figure 3b,c). The significant broadening and shift of the peaks of *ν*(O–H) from 3240 cm^−1^ to 3187 cm^−1^ and *ν*(C=O) from 1609 cm^−1^ shifted to 1599 cm^−1^ in resveratrol and oxymatrine could be identified in the co-amorphous OMAR-RES, as well as the decreased *ν*(N^+^–O^−^) signal in 2286 cm^−1^ of oxymatrine (Figure 3b, Figures S4 and S8). Similarly, the signals belonging to *ν*(O–H) of resveratrol and *ν*(C=O) of sophoridine at 3240 cm^−1^ and 1620 cm^−1^ were both shifted to 3188 cm^−1^ and 1590 cm^−1^, respectively (Figure 3c). Taken together, these data suggest that there are certain intermolecular interactions between the O–H groups of resveratrol and the C=O group of the three tested alkaloids, while additional interactions may exist between the phenolic groups of resveratrol and the *N*-oxide of oxymatrine.

### 3.4. The Molecular Interactions of Resveratrol and Oxymatrine in DMSO-d_6_

**Figure 4:**
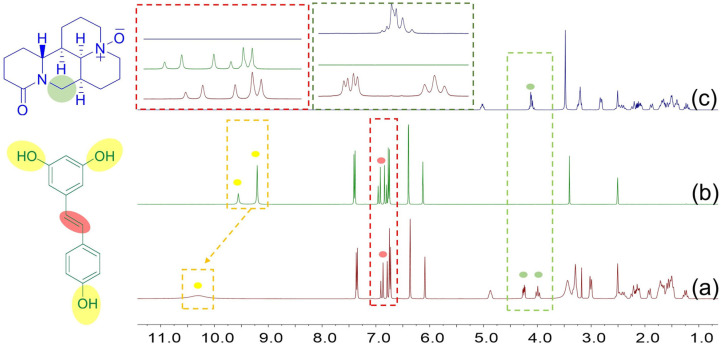
One-dimensional ^1^H NMR spectra of co-amorphous forms of OMAR-RES (a), resveratrol (b), and oxymatrine (c) dissolved in DMSO-*d*_6_.

The presence of molecular interactions in the above co-amorphous samples raised an interesting question of whether these interactions are also present in solution, which may affect their release behaviors and pharmacokinetics. Therefore, we opted to study co-amorphous MAR-RES, OMAR-RES, and SPD-RES in the aprotic solvent DMSO-*d*_6_, using ^1^H and ^13^C NMR spectroscopy (Figure 4 and Figures S11–S13 and Tables S2–S4). The reason is that many of these interactions involve hydrogen bond interactions including the phenolic groups of resveratrol and the carbonyl group of the lactam ring in these alkaloids, along with the *N*-oxide functional group of oxymatrine. The peak assignment of matrine, oxymatrine, sophoridine, and resveratrol in their ^1^H NMR and ^13^C NMR spectra was summarized in the supporting information Tables S2–S4 [41,42,43]. There were slight changes of certain chemical shifts in MAR-RES and SPD-RES samples in their NMR spectra in comparison to their individual counterparts, suggesting negligible interactions between MAR-RES and SPD-RES under the tested conditions (Figures S11 and S12, Table S2). In contrast, significant changes of certain chemical shifts in co-amorphous OMAR-RES were observed in both the ^1^H NMR and ^13^C NMR spectra, compared to their crystalline counterparts in DMSO-*d*_6_ (Figure 4 and Figure S13). For example, two series of phenolic groups of resveratrol were downfield shifted by 0.74 and 1.09 ppm to merge into a broad peak at *δ* = 10.3 ppm, suggesting a strong shielding effect probably by the formation of inter-molecular hydrogen bonds with oxymatrine, consistent with the downfield shifted signals of C-3, C-5, and C-4′ of resveratrol in the ^13^C NMR spectrum of OMAR-RES (Table S4). In addition, the downfield shifted signals of H-2 and H-6 (Δ*δ* = ~0.2 ppm) and C-6 (Δ*δ* = ~0.7 ppm), and the upfield shifted signals of C-2, C-8, and C-10 (Δ*δ* = ~0.6, 0.4, and 0.5 ppm) from oxymatrine strongly suggesting that the hydrogen bond acceptor is the *N*-oxide in oxymatrine. Taken together, the unique *N*-oxide in oxymatrine in OMAR-RES seemed to impose the important intermolecular interaction with resveratrol in DMSO-*d*_6_.

### 3.5. Dissolution Experiments

**Figure 5:**
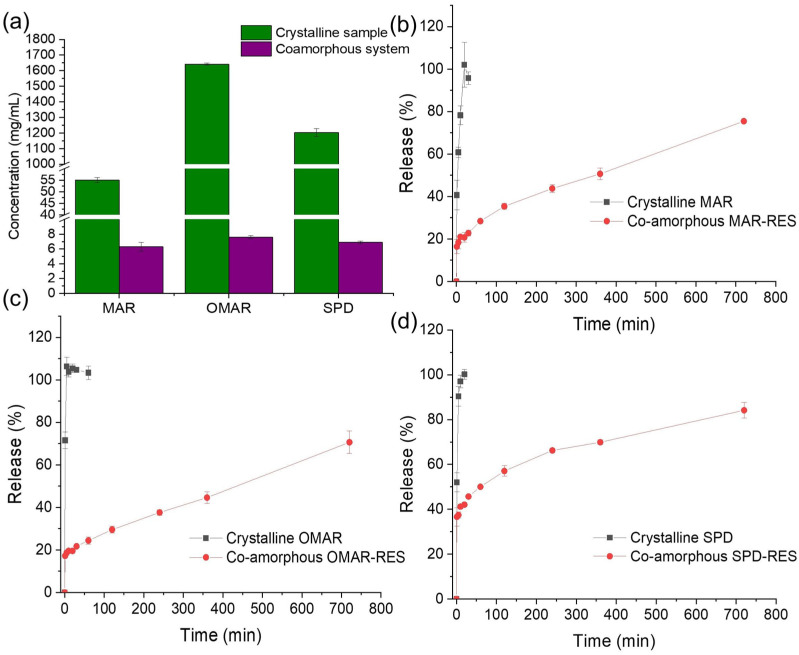
Equilibrium solubilities of matrine, oxymatrine, and sophoridine from their crystalline and co-amorphous forms (**a**); release profiles of MAR from crystalline MAR and co-amorphous MAR-RES (**b**); OMAR from crystalline OMAR and co-amorphous OMAR-RES (**c**); SPD from crystalline SPD, and co-amorphous SPD-RES (**d**) in pH 6.8 PBS at 37 ± 0.5 °C.

We next determined the equilibrium solubility of matrine, oxymatrine, sophoridine in their co-amorphous systems with resveratrol (Figure 5a). The equilibrium solubility of the crystalline forms of matrine, oxymatrine, sophoridine was 55, 1642, and 1203 mg/mL at 37 °C in PBS buffer (pH 6.8), while their individual one in the co-amorphous systems was drastically reduced to 6.3, 7.6, and 6.9 mg/mL, respectively. In contrast, no resveratrol could be observed under the current tested conditions, probably due to its poor water solubility. We further evaluated the residue solids after these equilibrium solubility tests using PXRD (Figures S14–S16). Interestingly, only the diffraction peaks of resveratrol emerged and no diffraction signals of matrine-type alkaloids could be observed on the PXRD patterns of the samples after dissolution tests. The diffraction peaks in Figure S16c are weaker than those of other two co-amorphous samples (Figures S14c and S15c) after dissolution and marked with asterisks for clarity. The recrystallization of resveratrol in the co-amorphous systems may be more significantly interfered with by sophoridine in comparison to the other two co-amorphous systems. The observed typical halo in PXRD suggests the presence of the remaining co-amorphous solids in the tested samples.

The in vitro release behaviors of the three co-amorphous systems and their crystalline counterparts were next studied (Figure 5b–d). Matrine, oxymatrine, and sophoridine displayed sustained release behavior from their co-amorphous forms, compared to the crystalline one. For example, the crystalline matrine released totally within 30 min, while only up to ~75% of matrine released from the co-amorphous MAR-RES after 12 h (Figure 5b). The crystalline oxymatrine and sophoridine also released rapidly and reached almost 100% cumulative release within 30 min (Figure 5c,d). However, ~71% of oxymatrine and ~84% of sophoridine released from their co-amorphous systems after 12 h. It appeared that oxymatrine released the slowest among the three co-amorphous systems, probably due to the stronger interactions between oxymatrine and resveratrol. Interestingly, sophoridine released the fastest from co-amorphous SPD-RES among the tested co-amorphous systems during the first 5 min, with ~35% released sophoridine into the solution, in comparison to ~20% released matrine or oxymatrine from their respective co-amorphous systems, which may be because sophoridine does not have such strong molecular interactions in the liquid state as oxymatrine and resveratrol. This result suggests that resveratrol might be an excellent co-former in co-amorphous drug systems for the sustained release of potential drugs. 

After the release experiments, the morphology of the residue tablets was examined, revealing that the surface and the inside of tablets were in two different states, since the tablet surface was brown-colored, dense, and easy to peel off. SEM study revealed that all of the residual tablets inside had round edges and smooth surfaces-like fragments, which were similar to the co-amorphous powder prepared by the same method (Figure S17a–c) [44]. In contrast, the SEM images of the tablet surface showed a rough, irregular, and angular morphology, suggesting the occurrence of recrystallization (Figure S17d–f), which was next confirmed to be crystalline resveratrol by PXRD analysis, while all of the interior tablets kept the amorphous solid state (Figures S18–S20). 

**Figure 6:**
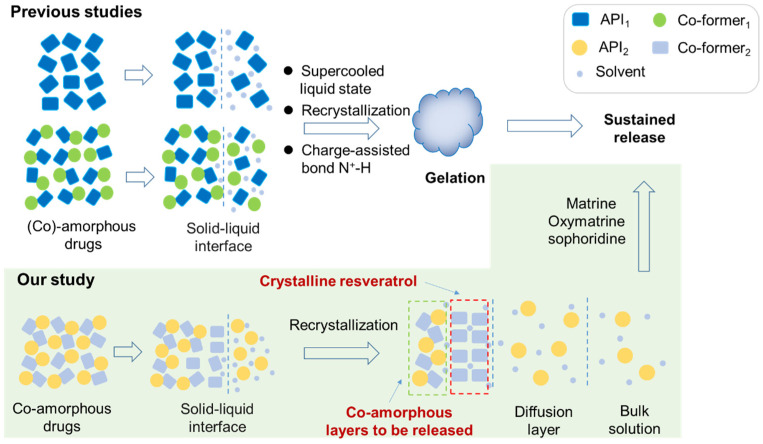
The proposed sustained-release mechanism for co-amorphous matrine/oxymatrine/sophoridine and resveratrol, in comparison to previously reported gelation-based release mechanism.

Therefore, we reasoned that there might be a distinct release mechanism of the three matrine-type alkaloids in their co-amorphous systems (Figure 6). The slow-release behavior due to gel formation has been studied in (co)-amorphous lurasidone hydrochloride, indomethacin/paracetamol, sinomenine with phenolic acids, and nonsteroidal anti-inflammatory drugs [28,32,33]. Qian and co-workers proposed that the gel formation was mediated by charge-assisted hydrogen bond formation [30]. In contrast, we discovered that the highly soluble alkaloids, e.g., matrine, rapidly dissolved from the tablet surface, resulting in the accumulation of an abundant amount of recrystallized resveratrol on the outer layer of tablets in contact with the aqueous solution (Figure S17). However, the interior portions of the tablets remained in an amorphous state (Figures S18d–S20d). The observed sustained release of matrine, oxymatrine, and sophoridine is likely because the recrystallization of resveratrol significantly limited the access of the PBS buffer to the co-amorphous systems inside the tablets (Figures S17–S20). The crystallized resveratrol on the surface of co-amorphous samples during the dissolution process may have formed a “shell-like” structure for the sustained release of matrine-type alkaloids.

### 3.6. Physical Stability

The physical stability of the three co-amorphous drug systems were evaluated under three conditions (i.e., 25 °C, 40 °C/low RH, or 25 °C/75% RH) (Figures S21 and S22). Under 25 °C/low RH condition, all the tested co-amorphous samples maintained their amorphous solid-state after nine months of storage (Figure S21). Under accelerated storage condition (40 °C/low RH or 25 °C/75% RH), no diffraction peaks were found in these samples after 4 or 2 months, respectively (Figure S22). This result suggests that the tested co-amorphous samples possess excellent physicochemical stability under all experimental conditions. 

In order to study if recrystallization of the three co-amorphous samples occurred after compression, the compressed samples were next investigated by PXRD. As shown in Figure S23, no diffraction peaks appeared in the PXRD patterns of all the tested co-amorphous samples after compressed under the pressure of 937.5 MPa, suggesting their promising mechanical stability.

## 4. Conclusions

Repurposing known drugs is an efficient approach to the fight against SARS-CoV-2. Based on the encouraging observational study of matrine for COVID-19, we prepared and evaluated the MAR-RES co-amorphous drug system, together with OMAR-RES and SPD-RES. We discovered that the three co-amorphous systems showed sustained release behavior, enabling the future development and clinical evaluation of a more convenient oral delivery route than continuous infusion for these excellent drug candidates against SARS-CoV-2. The crystallized resveratrol on the surface of co-amorphous samples during the dissolution process formed a “shell-like” structure, which may hinder the release of matrine-type alkaloids and realizes the sustained release. In addition, the similar yet structurally distinct interactions of matrine, oxymatrine, and sophoridine with the co-former resveratrol suggest that intermolecular interactions play a prominent role in their release. Considering the salient biological activity of resveratrol, it may be used as a new type of drug co-former in the preparation of co-amorphous drug samples. However, the recrystallization of resveratrol prevents its release under the tested conditions, resulting in no solubility improvement. Therefore, further studies are also needed to improve the release behavior of resveratrol, as well as the pharmacokinetics and therapeutic effects of the above co-amorphous systems in animal models. 

## Data Availability

Not applicable.

## Supplementary Materials

The following supporting information can be downloaded at: https://www.mdpi.com/article/10.3390/pharmaceutics14030603/s1. Table S1: The commercial drug formulations of matrine, oxymatrine, and sophoridine on the markets in China. Tables S2 and S3: The ^1^H NMR chemical shifts changes of MAR, SPD, OMAR and RES in DMSO-*d*_6_ after co-amorphization. Table S4: The ^13^C NMR chemical shifts changes of OMAR and RES in DMSO-*d*_6_ after co-amorphization. Figure S1: PXRD patterns of raw material OMAR (a), simulated of OMAR monohydrate (b) and OMAR trihydrate (c) derived from the single crystal structure. Figure S2: PXRD patterns of crystalline samples and the sample after rotary evaporation. Figures S3–S10: FTIR spectra of crystalline MAR, OMAR, SPD, the three co-amorphous forms, and their physical mixtures. Figures S11 and S12: The comparison of ^1^H NMR spectra of the co-amorphous forms of MAR-RESand SPD-RES dissolved in DMSO-*d*_6_. Figure S13: The comparison of ^13^C NMR spectra of co-amorphous forms of OMAR-RES dissolved in DMSO-*d*_6_. Figures S14–S16: PXRD patterns of three co-amorphous forms after the equilibrium solubility test. Figure S17: SEM images of the powder samples. Figures S18–S20: PXRD patterns of three co-amorphous forms after release tests. Figure S21: PXRD patterns of three CASs. Figure S22: The physicochemical stability of the three co-amorphous systems. Figure S23: PXRD patterns of three co-amorphous systems after pressure tests.
