# Supplementary material for: Sustained Release of Co-Amorphous Matrine-Type Alkaloids and Resveratrol with Anti-COVID-19 Potential
Source: Pharmaceutics. 2022 Mar 10;14(3):603. doi: 10.3390/pharmaceutics14030603 (PMC8949968; doi:10.3390/pharmaceutics14030603)
Supplement: Supplementary file 1 [file pharmaceutics-14-00603-s001.zip › pharmaceutics-1577902-supplementary.pdf]

pharmaceutics

Supplementary Materials: Sustained Release of Co-Amorphous Matrine-Type Alkaloids and Resveratrol with Anti-COVID-19 Potential

Dandan Hu, Xin Chen, Duanxiu Li, Hailu Zhang, Yanwen Duan and Yong Huang

Table S1. The commercial drug formulations of matrine, oxymatrine, and sophoridine on the markets in China.

Product	Dosage form	APIs	Indications	Specifications (mg)
Alkaloidi sophorae tannate tablets	Tablet	Matrine	Enteritis	10
Sophora alkaloids tannate capsules	Capsule	Matrine	Enteritis	44
Matrine and glucose injection	Injection	Matrine	Chronic hepatitis	150
Matrine and sodium chloride injection	Injection	Matrine	Chronic hepatitis	80
Matrine vaginal effervescent tablets	Tablet	Matrine	Antibacterial blend	50
Matrine vaginal expansion suppository	Suppository	Matrine	Antibacterial blend	50
Sophora gel	gel	Matrine	Antibacterial blend	100
Hepatitis ling injection	Injection	Matrine	For chronic, active hepatitis	35
Marine tablets	Tablet	Oxymatrine	Chronic hepatitis	100
Marine dispersible tablets	Tablet	Oxymatrine	Chronic hepatitis	100
Marine for injection	Injection	Oxymatrine	Chronic hepatitis, Tumor chemotherapy	300/600
Marine injection	Injection	Oxymatrine	Chronic hepatitis, Tumor chemotherapy	200/600
Marine and sodium chloride injection	Injection	Oxymatrine	Chronic hepatitis	600
Marine and glucose injection	Injection	Oxymatrine	Chronic hepatitis	200
Sophoridine hydrochloride injection	Injection	Sophoridine	Tumor chemotherapy	25

The data are adpated from National Medical Products Administration (https://www.nmpa.gov.cn/index.html). (accessed on October 1, 2021).

Table S2. The ^{1}H NMR chemical shifts changes of MAR, SPD and RES in DMSO-d_6 after co-amorphization.

Position	Chemical Shifts (δ, ppm)						
	RES	MAR	SPD	MAR-RES		SPD-RES	
	δ	δ	Δ	δ	Δδ	δ	Δδ
4′-OH	9.56	-	-	9.55	-0.01	9.55	-0.01
3-OH/5-OH	9.21	-	-	9.20	-0.01	9.20	-0.01
H-2′/H-6′	7.40	-	-	7.39	-0.01	7.39	-0.01
H-α′	6.94	-	-	6.93	-0.01	6.93	-0.01
H-α	6.82	-	-	6.81	-0.01	6.81	-0.01
H-3′/H-5′	6.76	-	-	6.75	-0.01	6.75	-0.01
H-2/H-6	6.39	-	-	6.38	-0.01	6.38	-0.01
H-4	6.13	-	-	6.11	-0.02	6.11	-0.02
H-17a		4.19	m	4.18	-0.01	m	-
H-11		3.72	m	3.71	-0.01	m	-

$\Delta\delta = \delta_{\text{(co-amorphous systems)}} - \delta_{\text{(RES, MAR or SPD)}}$, Negative Δδ means shift upfield, vice versa.

Table S3. The ^{1}H NMR chemical shifts changes of OMAR and RES in DMSO-d_6 after co-amorphization.

Position	RES	OMAR	OMAR-RES	
	**Chemical Shifts (δ, ppm)**			
	δ	*Δ*	Δ*δ*	Δ*δ*
4′-OH	9.56	-	10.30	+0.74
3-OH/5-OH	9.21	-	10.30	+1.09
H-2′/H-6	7.40	-	7.36	-0.04
H-α′	6.94	-	6.89	-0.05
H-α	6.82	-	6.77	-0.05
H-3′/H-5′	6.76	-	6.74	-0.02
H-2/H-6	6.39	-	6.36	-0.03
H-4	6.13	-	6.09	-0.04
H-11	-	5.02	4.88	-0.14
H2-17	-	4.11	d	+0.14/-0.12
H2-10/H-2a	-	3.20	3.28	+0.08
H-2b/H-6	-	2.82	3.02	+0.20

Δ*δ* = *δ* (co-amorphous systems) – *δ* (RES or OMAR). Negative Δ*δ* means shift upfield, vice versa.

Table S4. The ^{13}C NMR chemical shifts changes of OMAR and RES in DMSO-*d6* after co-amorphization.

Position	RES	OMAR	OMAR-RES	
	**Chemical Shifts (δ, ppm)**			
	δ	Δ	Δ*δ*	Δ*δ*
C-3/C-5	158.9	-	159.4	+0.5
C-4′	157.7	-	158.3	+0.6
C-1	139.6	-	139.5	-0.1
C-1′	128.5	-	128.2	-0.3
C-α′	128.4	-	128.1	-0.3
C-2′/C-6′	128.3	-	128.0	-0.3
C-α	126.1	-	126.0	-0.1
C-2/C-6	104.8	-	104.5	-0.3
C-4	102.2	-	102.3	-0.1
C-15	-	169.0	169.2	+0.2
C-2	-	68.8	68.2	-0.6
C-10	-	68.3	67.8	-0.5
C-6	-	65.4	66.1	+0.7
C-11	-	53.0	53.3	+0.3
C-7	-	42.2	41.9	-0.3
C-5	-	34.4	34.2	-0.2
C-4	-	26.1	25.8	-0.3
C-8	-	24.5	24.1	-0.4

Δ*δ* = *δ* (co-amorphous systems) – *δ* (RES or OMAR). Negative Δ*δ* means shift upfield, vice versa.

The ^{1}H and ^{13}C NMR chemical shifts of MAR, OMAR, SPD and RES.

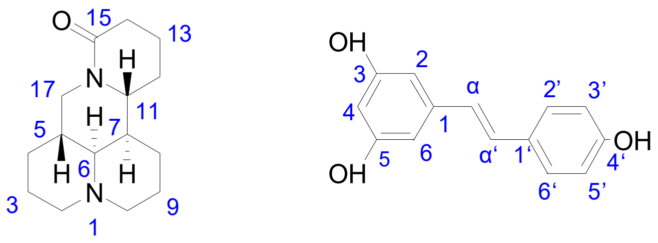

Matrine (MAR)[a]. ^{1}H NMR (400MHz, DMSO-d_6, δ, ppm, *J*/Hz): 4.19 (1H, dd, J=12.7, 4.1, H-17a), 3.72 (1H, m, H-11), 2.91 (1H, t, J=12.7, H-17b), 2.73 (2H, m, H-2a, H-10a), 2.24–1.82(7H, m), 1.75–1.25 (12H, m).

Oxymatrine (OMAR)[b]. ^{1}H NMR (400MHz, DMSO-d_6, δ, ppm, *J*/Hz): 5.02 (1H, m, H-11), 4.11 (2H, m, H2-17), 3.20 (3H, m, H2-10, H-2a), 2.82 (2H, m, H-2b, H-6), 2.49-1.85 (6H, m), 1.75-1.17(10H, m). ^{13}C NMR (100MHz, DMSO-*d6*, δ, ppm): 169.0 (C-15), 68.8 (C-2), 68.3 (C-10), 65.4 (C-6), 52.9 (C-11), 42.1 (C-7), 41.6 (C-17), 34.4 (C-5), 33.1 (C-14), 28.2 (C-12), 26.1 (C-4), 24.5 (C-8), 19.0 (C-13), 17.3 (C-3, C-9).

Sophoridine (SPD)[a]. ^{1}H NMR (400MHz, DMSO-d_6, δ, ppm, *J*/Hz): 3.34-3.20 (2H, m, H-11, H-17a), 3.07 (1H, m, H-17b), 2.77-2.62 (2H, m, H-10a, H-2a), 2.18-1.92 (5H, m), 1.87-1.67 (6H, m), 1.59-0.88 (8H, m).

Resveratrol (RES)[C]. ^{1}H NMR (400MHz, DMSO-d_6, δ, ppm, *J*/Hz): 9.56 (1H, s, 4′-OH), 9.21 (2H, s, 3-OH, 5-OH), 7.40 (2H, d, H-2′, H-6′), 6.94 (1H, d, H-α'), 6.82 (1H, d, H-α), 6.76 (2H, d, H-3′, H-5′), 6.39 (2H, d, H-2, H-6), 6.13 (1H, t, H-4). ^{13}C NMR (100MHz, DMSO-*d6*, δ, ppm): 158.9 (C-3, C-5), 157.7 (C-4′), 139.7 (C-1), 128.5 (C-1′), 128.4 (C-α'), 128.3 (C-2′, C-6′), 126.1 (C-α), 11 (C-3′, C-5′), 104.8 (C-2, C-6), 102.3 (C-4).

[a] According to ref. (Lei et al., 2014), [b] According to ref. (Hwang et al., 2005), [C] According to ref. (Pineda-Sanabria et al., 2011).

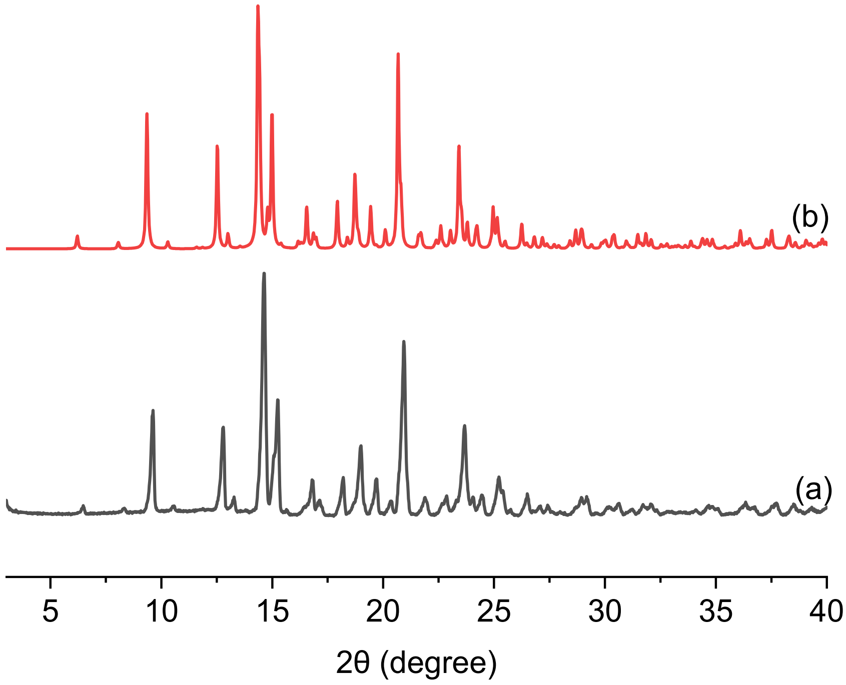

Figure S1. PXRD patterns of raw material OMAR (a), simulated of OMAR trihydrate (b) derived from the single crystal structure.

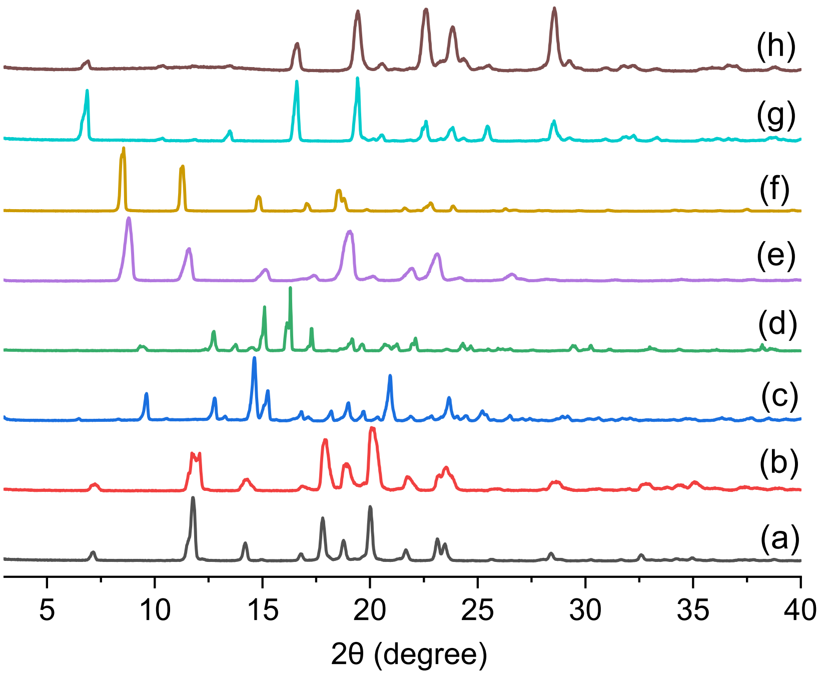

Figure S2. PXRD patterns of crystalline MAR (a), MAR after rotary evaporation (b); crystalline OMAR (c), OMAR after rotary evaporation (d); crystalline SPD (e), SPD after rotary evaporation (f); crystalline RES (g), RES after rotary evaporation (h).

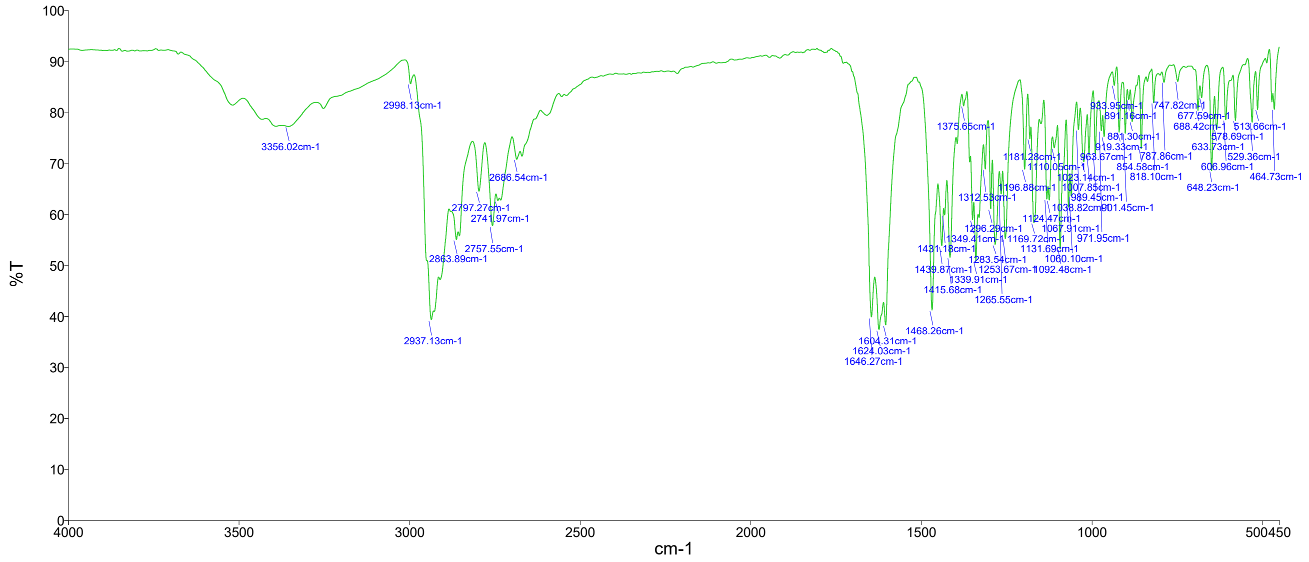

Figure S3. FTIR spectrum of crystalline MAR.

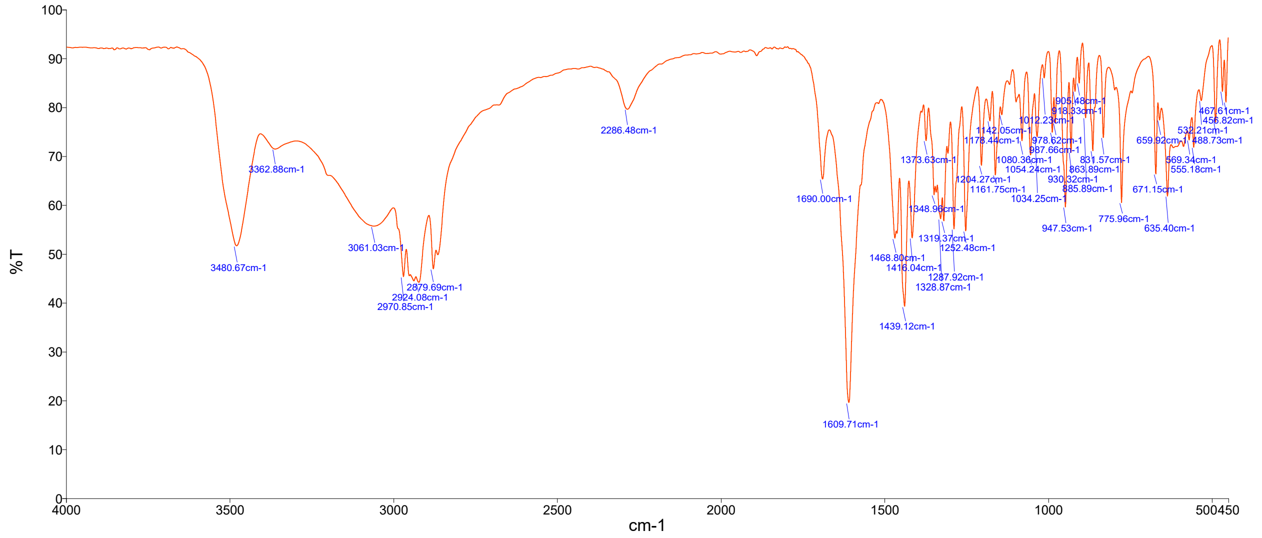

Figure S4. FTIR spectrum of crystalline OMAR.

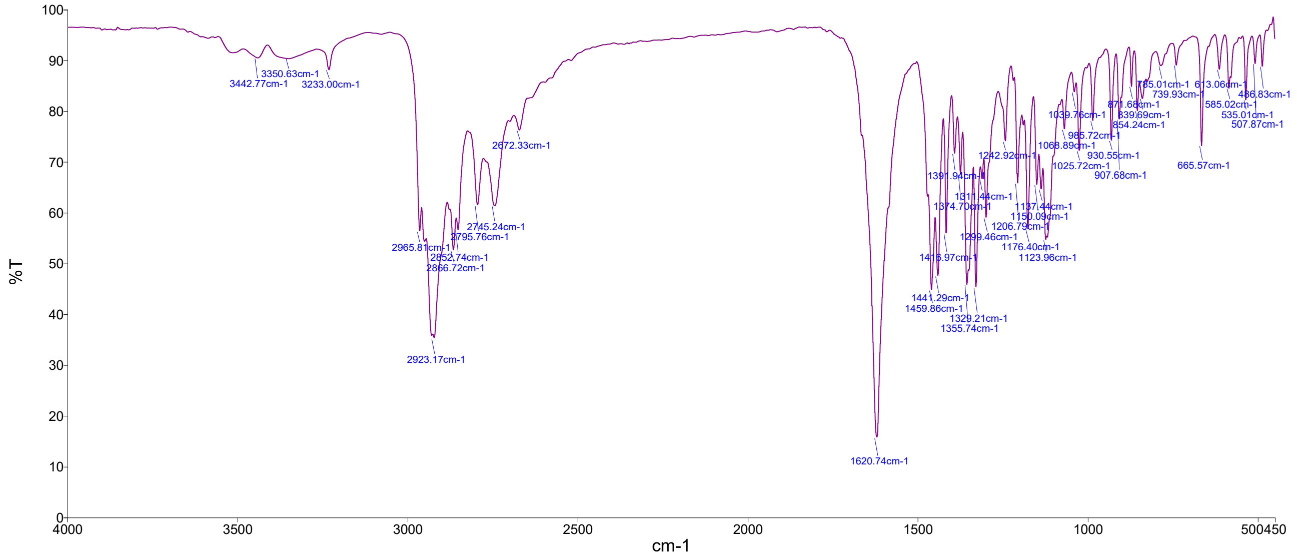

Figure S5. FTIR spectrum of crystalline SPD.

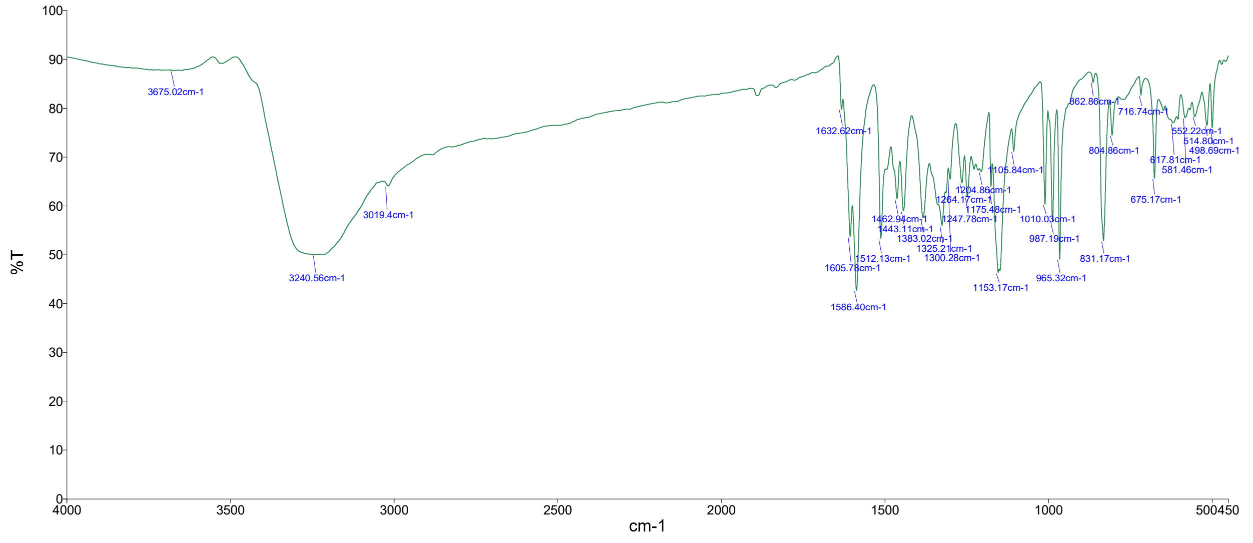

Figure S6. FTIR spectrum of crystalline RES.

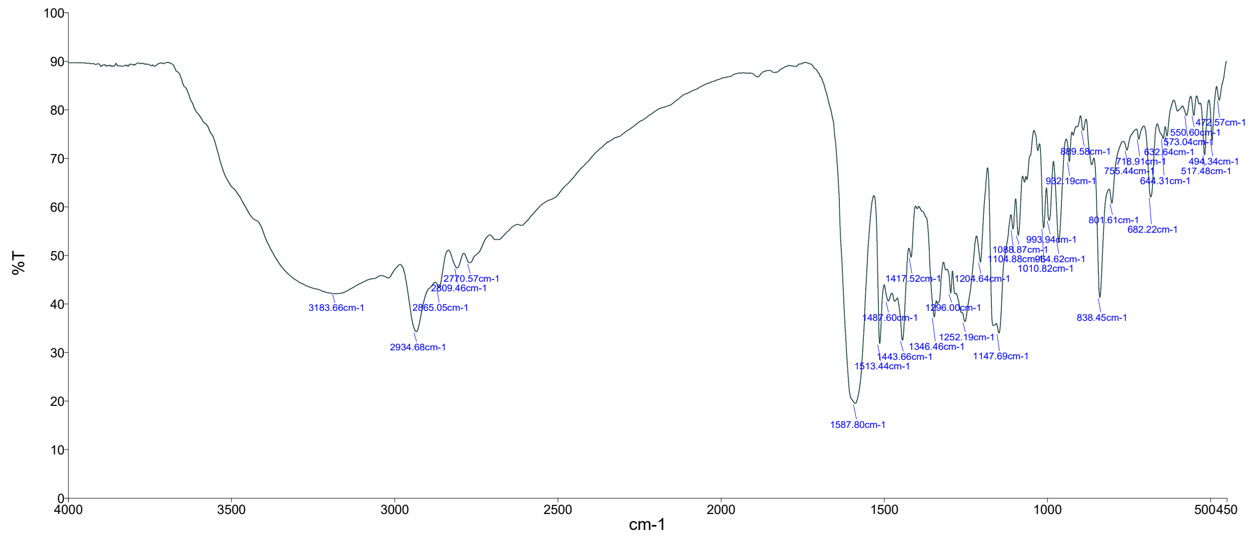

Figure S7. FTIR spectrum of co-amorphous forms of MAR-RES.

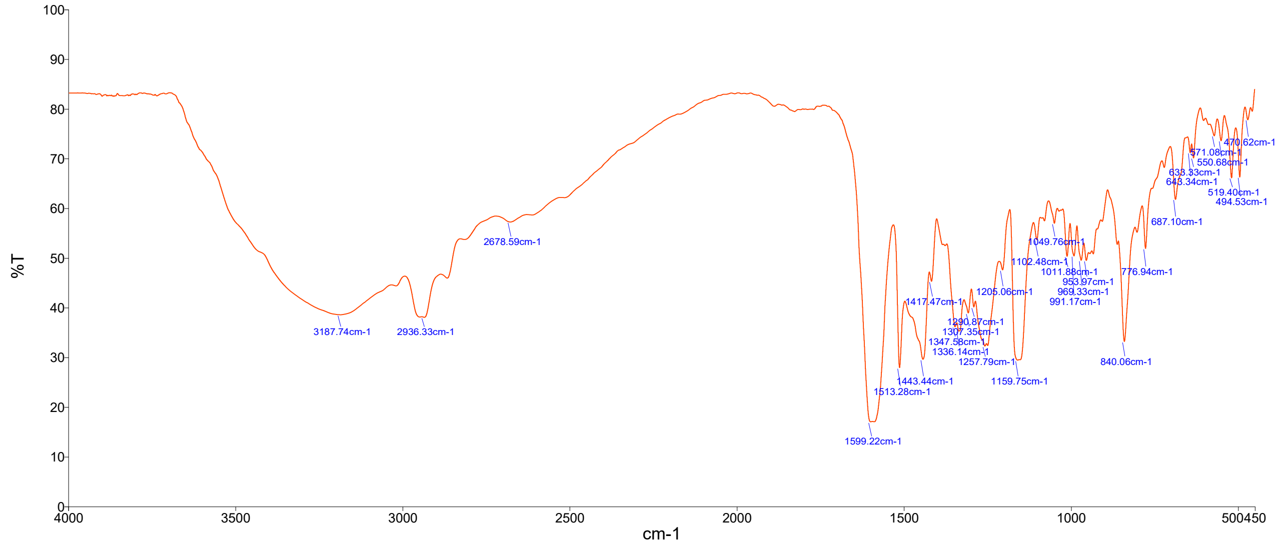

Figure S8. FTIR spectrum of co-amorphous forms of OMAR-RES.

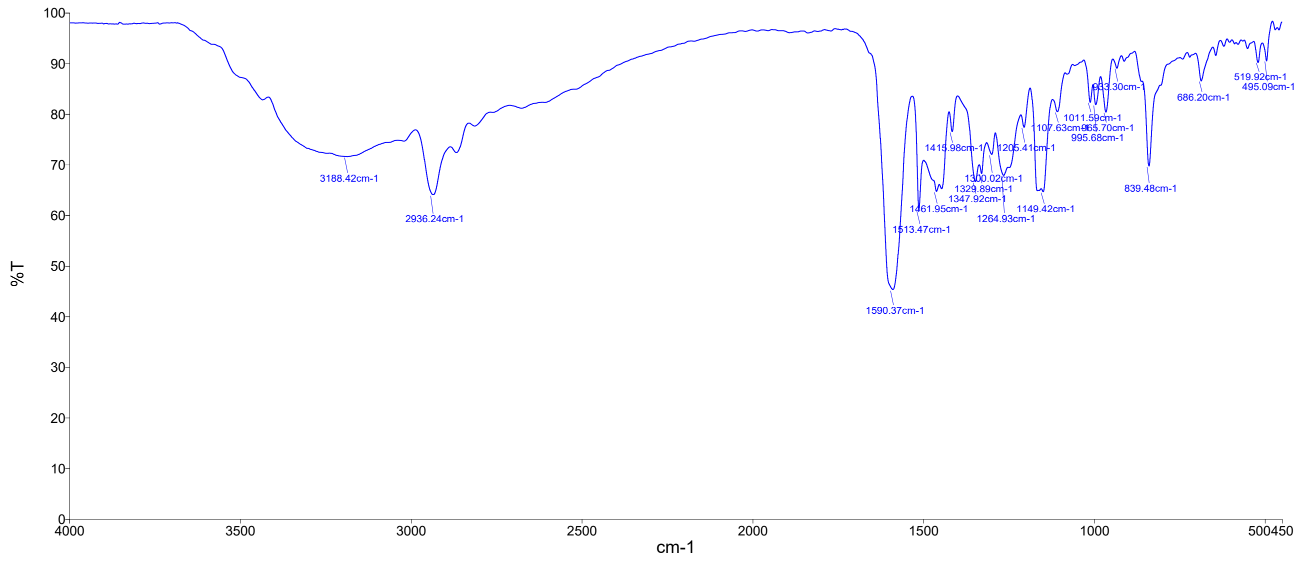

Figure S9. FTIR spectrum of co-amorphous forms of SPD-RES.

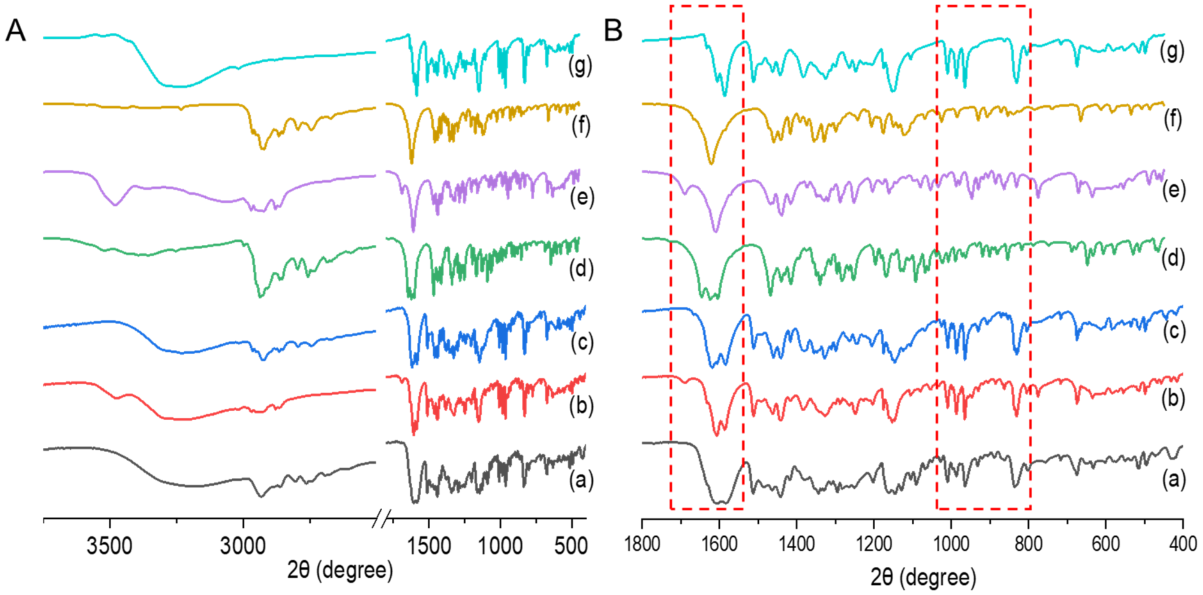

Figure S10. FTIR spectrum of physical mixture of crystalline MAR and RES (a), OMAR and RES (b), SPD and RES (c); crystalline MAR (d), OMAR (e), SPD (f), and RES (g) (full spectra (A) and partial enlargement spectra over 1800 to 400 cm^{-1} (B)).

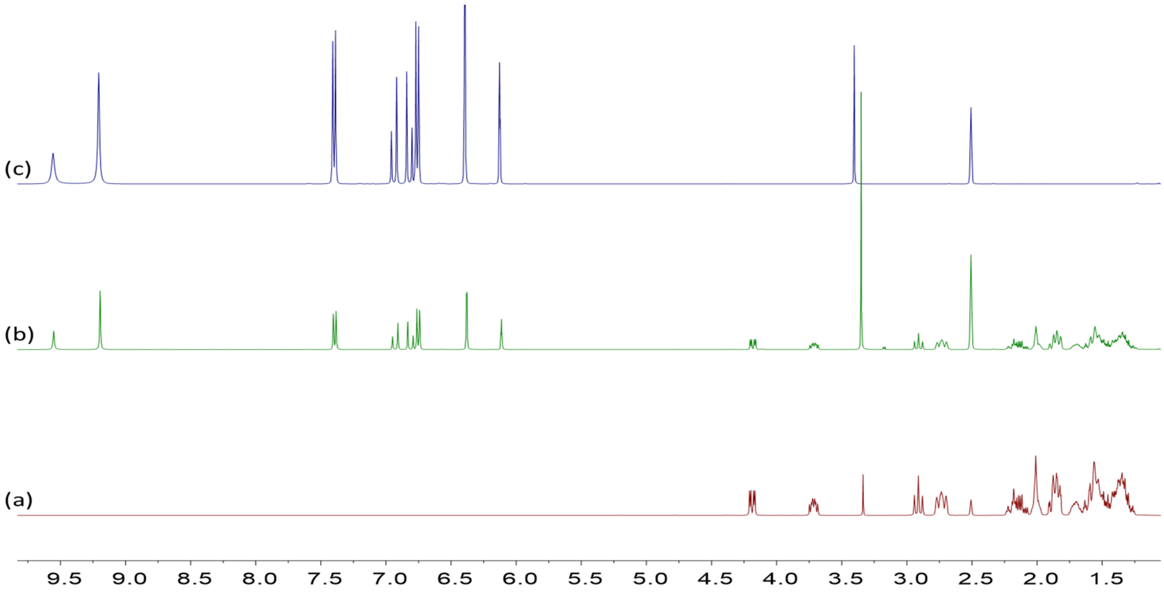

Figure S11. The comparison of ^{1}H NMR spectra of the co-amorphous forms of MAR-RES (b) with MAR (a) and RES (c) dissolved in DMSO-d_6.

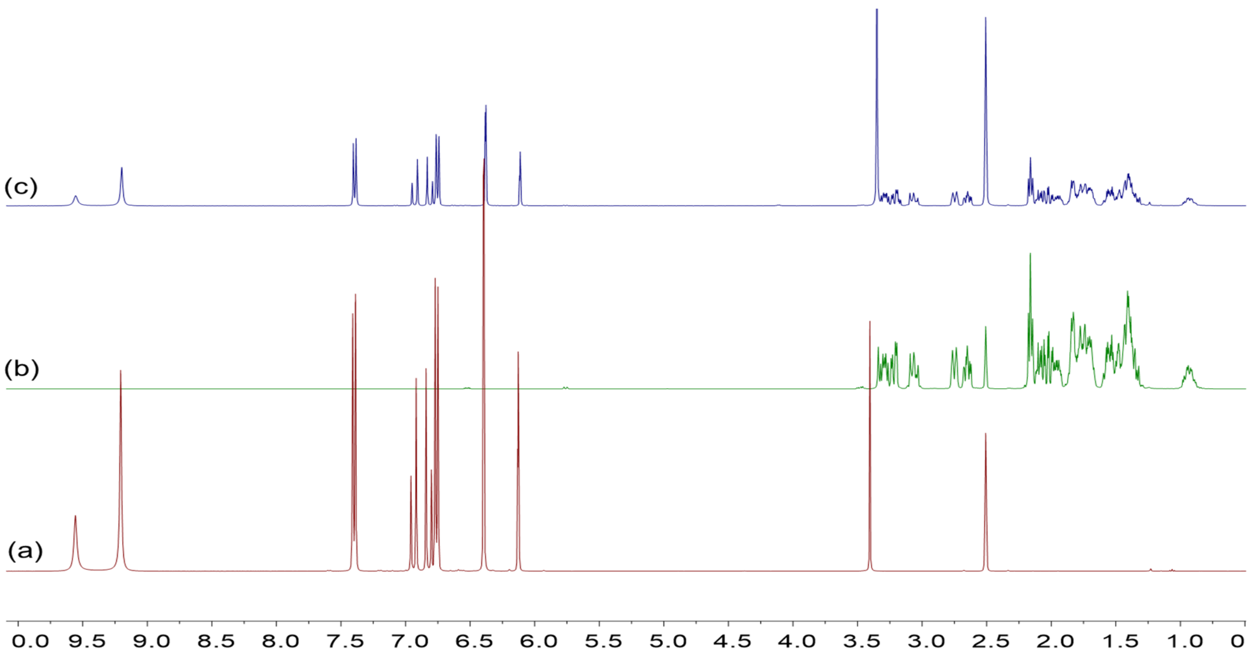

Figure S12. The comparison of ^{1}H NMR spectra of co-amorphous forms of SPD-RES (c) with RES(a) and SPD (b) dissolved in DMSO-d_6.

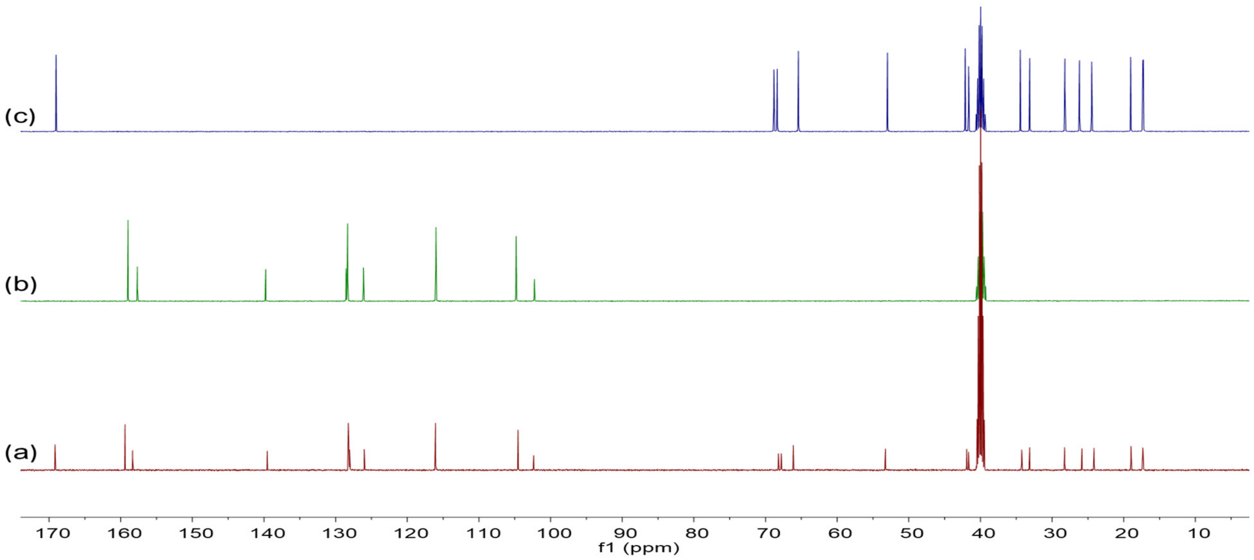

Figure S13. The comparison of ^{13}C NMR spectra of co-amorphous forms of OMAR-RES. (a) with RES (b) and OMAR (c) dissolved in DMSO-d_6.

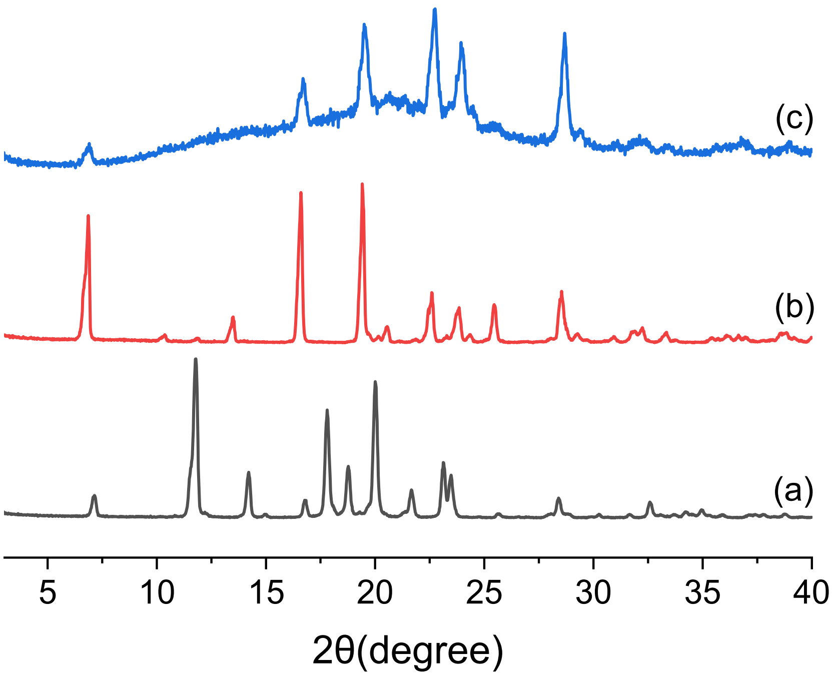

Figure S14. PXRD patterns of co-amorphous forms of MAR-RES powder after the equilibrium solubility test. (a) crystalline MAR, (b) crystalline RES, (c) co-amorphous MAR-RES powder after test.

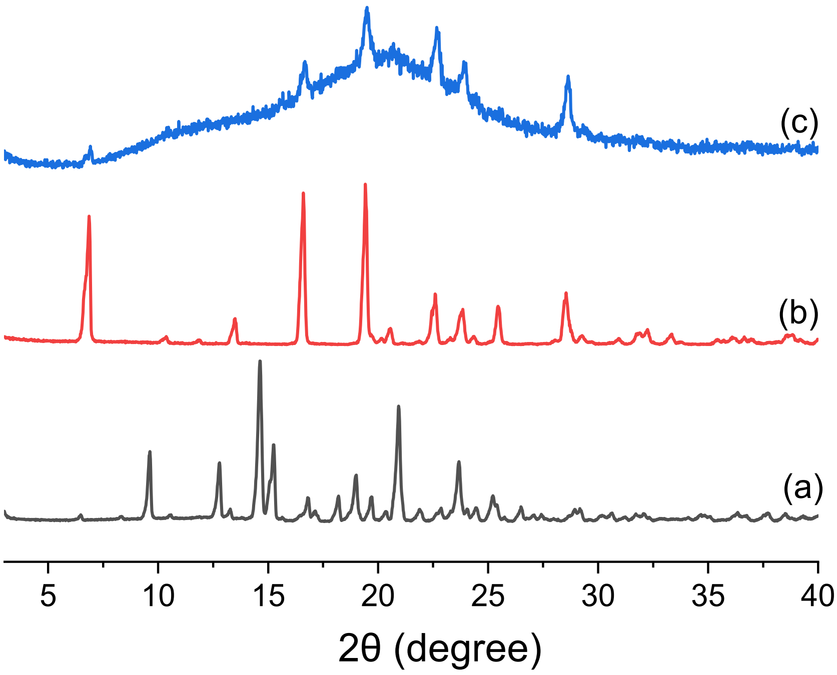

Figure S15. PXRD patterns of co-amorphous forms of OMAR-RES powder after the equilibrium solubility test. (a) crystalline OMAR, (b) crystalline RES, (c) co-amorphous OMAR-RES powder after test.

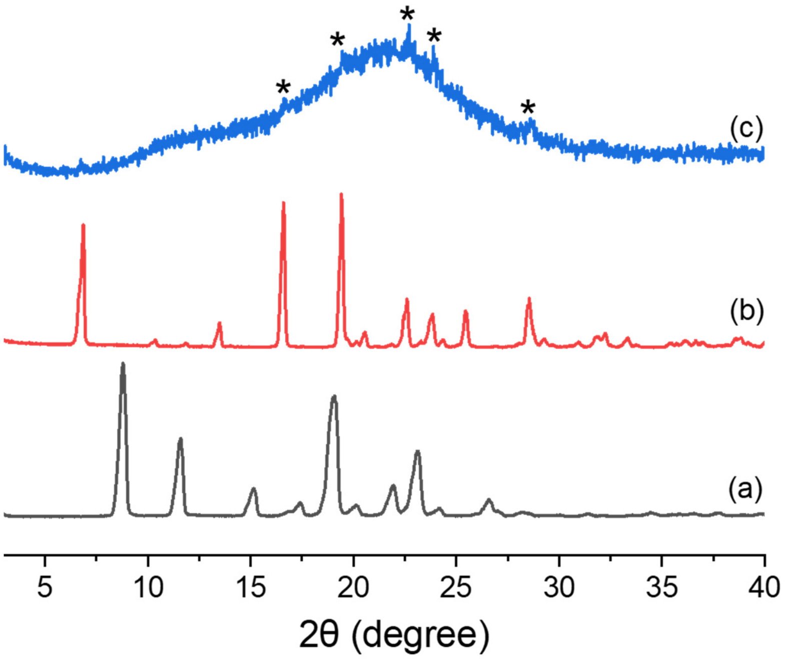

Figure S16. PXRD patterns of co-amorphous forms of SPD-RES powder after the equilibrium solubility test. (a) crystalline SPD, (b) crystalline RES, (c) co-amorphous SPD-RES powder after test.

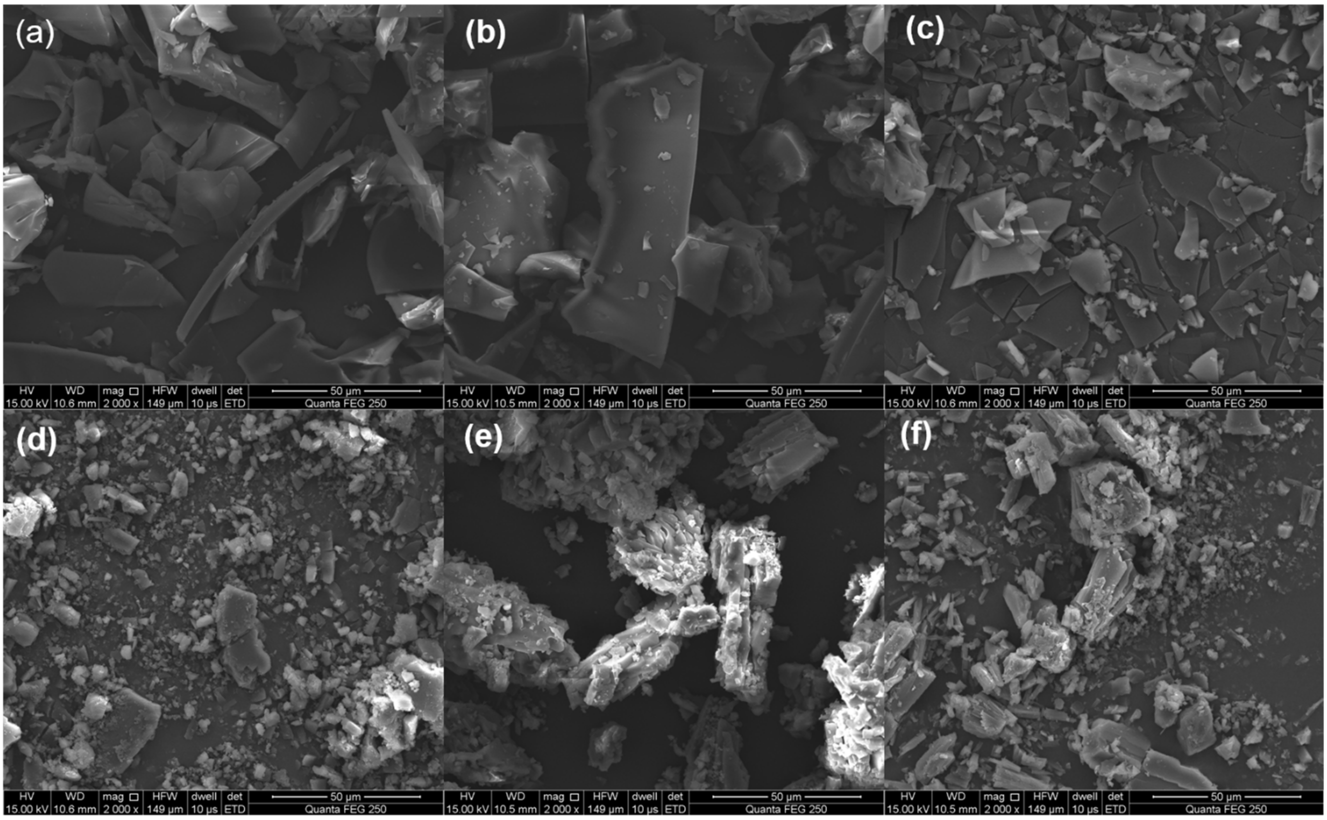

Figure S17. SEM images of the powder samples of fresh co-amorphous MAR-REA (a), OMAR-REA (b), SPD-RES (c), and the powder samples peeled from tablet surface after release tests of MAR-RES (d), OMAR-RES (e), and SDP-RES (f).

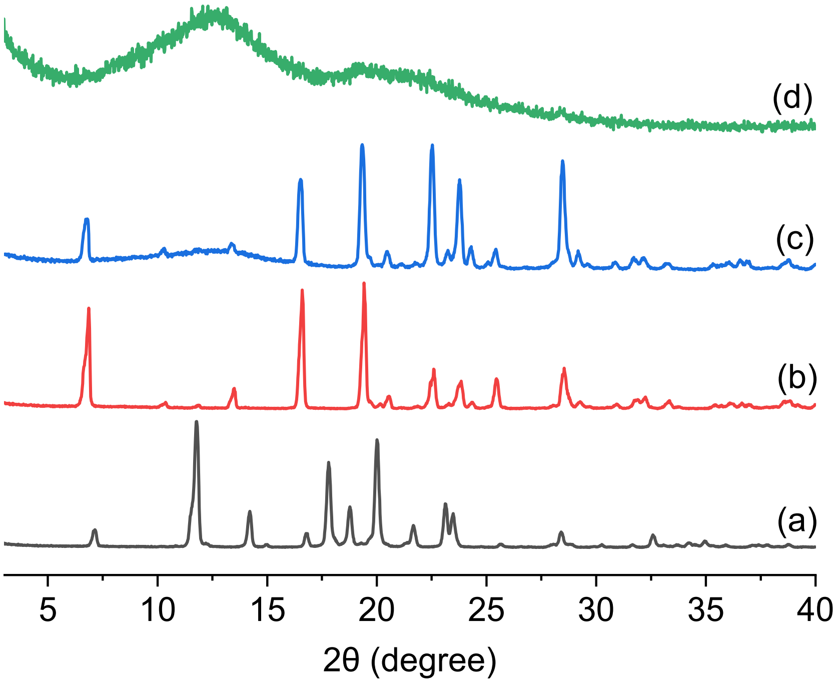

Figure S18. PXRD patterns of co-amorphous forms of MAR-RES after release tests. Raw material: (a) MAR, (b) RES; tablet after release: (c) MAR-RES-surface, (d) MAR-RES-inside.

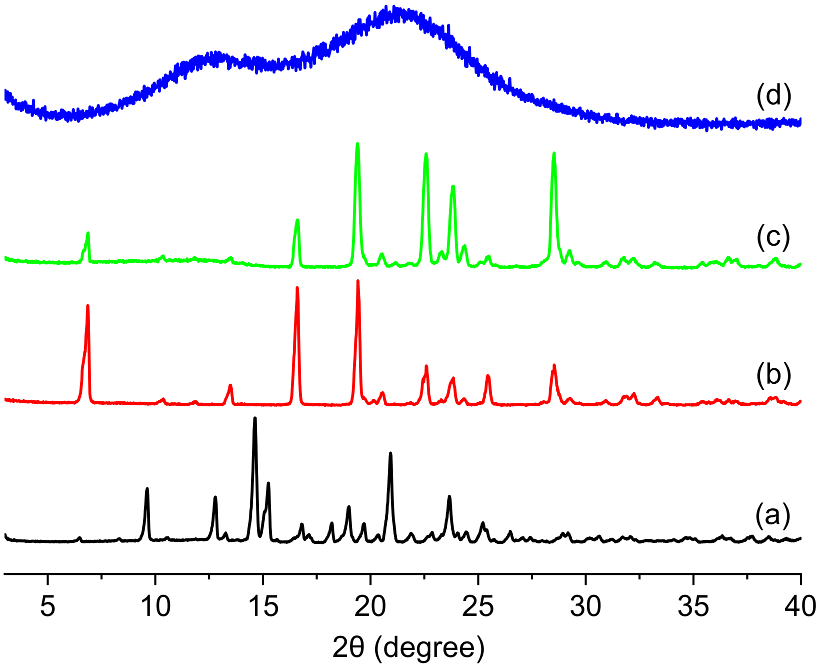

Figure S19. PXRD patterns of co-amorphous forms of OMAR-RES after release tests. Raw material: (a) OMAR, (b) RES; tablet after release: (c) OMAR-RES-surface, (d) OMAR-RES-inside.

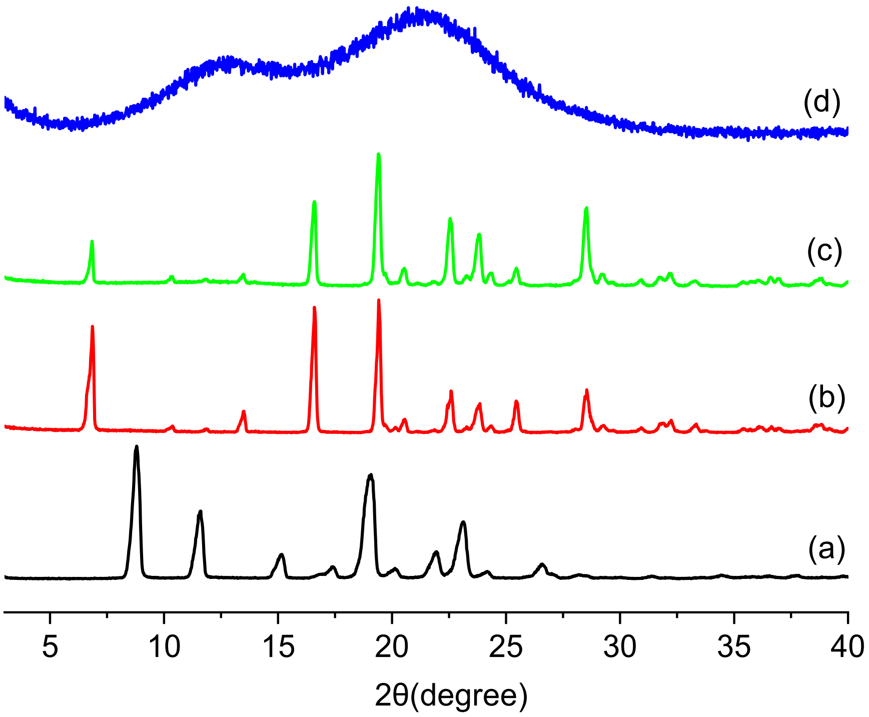

Figure S20. PXRD patterns of co-amorphous forms of SPD-RES after release tests. Raw material: (a) SPD, (b) RES; tablet after release: (c) SPD-RES-surface, (d) SPD-RES-inside.

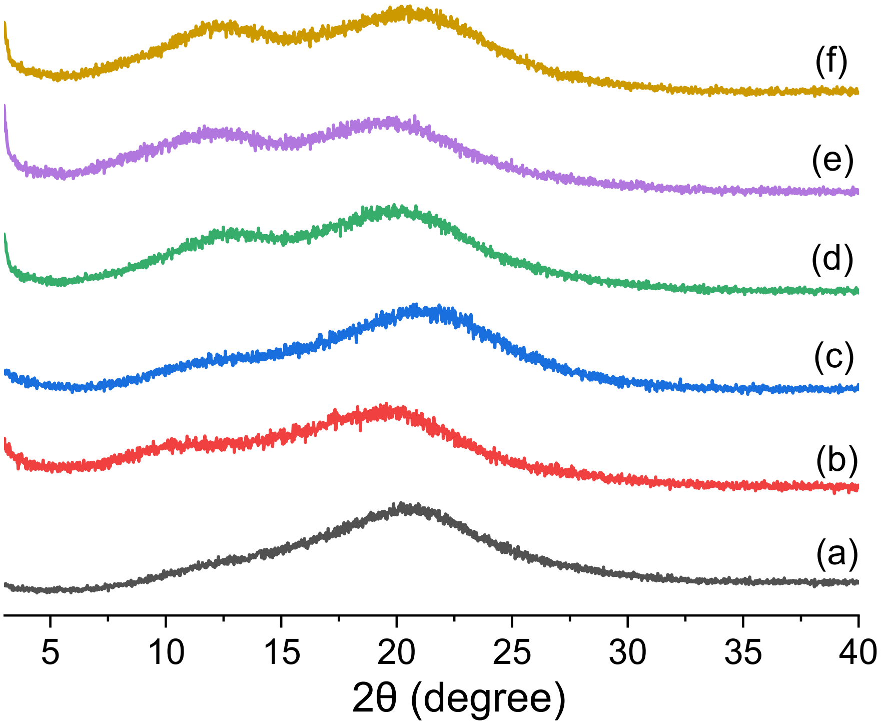

Figure S21. PXRD patterns of three CASs: MAR-RES fresh (a), OMAR-RES fresh (b), SPD-RES fresh (c), MAR-RES nine months (d), OMAR-RES nine months (e), SPD-RES nine months (f) under 25 ◦C/low RH.

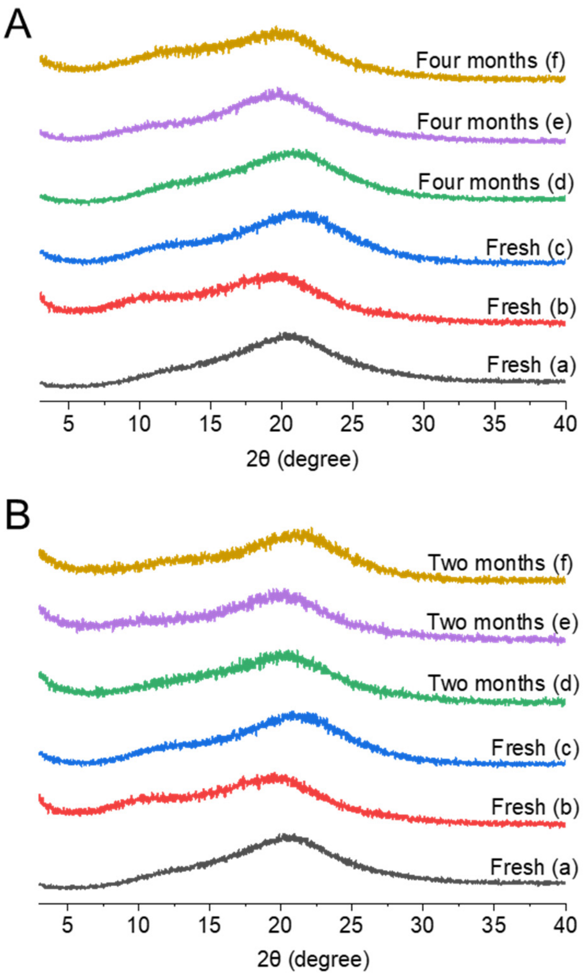

Figure S22. The physicochemical stability of the three co-amorphous systems. MAR-RES (a, d), OMAR-RES (b, e), and SPD-RES (c, f) before and after stored at different period at 40 °C /low RH (A) and 25 °C /75% RH (B).

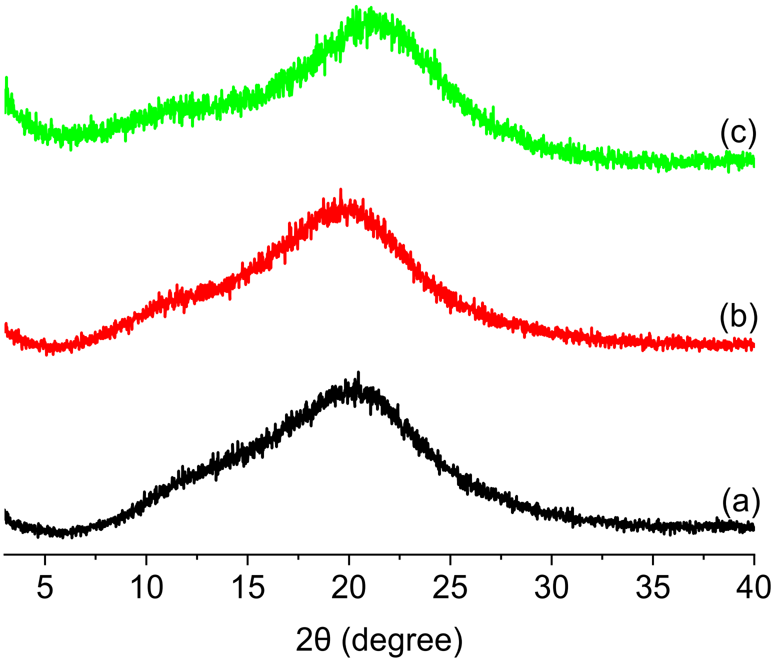

Figure S23. PXRD patterns of three co-amorphous systems after pressure tests. (a) MAR-RES, (b) OMAR-RES, (c) SPD-RES.
